# Rediscovering Beta-2 Microglobulin As a Biomarker across the Spectrum of Kidney Diseases

**DOI:** 10.3389/fmed.2017.00073

**Published:** 2017-06-15

**Authors:** Christos P. Argyropoulos, Shan Shan Chen, Yue-Harn Ng, Maria-Eleni Roumelioti, Kamran Shaffi, Pooja P. Singh, Antonios H. Tzamaloukas

**Affiliations:** ^1^Nephrology Division, Department of Internal Medicine, University of New Mexico School of Medicine, Albuquerque, NM, United States; ^2^Raymond G. Murphy VA Medical Center Albuquerque, Albuquerque, NM, United States

**Keywords:** beta-2 microglobulin, chronic kidney disease, biomarkers, kidney transplantation, pediatric nephrology, acute kidney injury, multiple myeloma, glomerular filtration rate

## Abstract

There is currently an unmet need for better biomarkers across the spectrum of renal diseases. In this paper, we revisit the role of beta-2 microglobulin (β_2_M) as a biomarker in patients with chronic kidney disease and end-stage renal disease. Prior to reviewing the numerous clinical studies in the area, we describe the basic biology of β_2_M, focusing in particular on its role in maintaining the serum albumin levels and reclaiming the albumin in tubular fluid through the actions of the neonatal Fc receptor. Disorders of abnormal β_2_M function arise as a result of altered binding of β_2_M to its protein cofactors and the clinical manifestations are exemplified by rare human genetic conditions and mice knockouts. We highlight the utility of β_2_M as a predictor of renal function and clinical outcomes in recent large database studies against predictions made by recently developed whole body population kinetic models. Furthermore, we discuss recent animal data suggesting that contrary to textbook dogma urinary β_2_M may be a marker for glomerular rather than tubular pathology. We review the existing literature about β_2_M as a biomarker in patients receiving renal replacement therapy, with particular emphasis on large outcome trials. We note emerging proteomic data suggesting that β_2_M is a promising marker of chronic allograft nephropathy. Finally, we present data about the role of β_2_M as a biomarker in a number of non-renal diseases. The goal of this comprehensive review is to direct attention to the multifaceted role of β_2_M as a biomarker, and its exciting biology in order to propose the next steps required to bring this recently rediscovered biomarker into the twenty-first century.

## Introduction

Chronic kidney disease (CKD) is a common public health issue associated with astonishingly high cardiovascular (CV) morbidity and mortality and high costs, particularly for patients with diabetic nephropathy. Patients with renal failure on maintenance dialysis have excess mortality, that is, eight times higher than that of the general population (1). Most patients die due to CV events related to both traditional and non-traditional risk factors (2) and this is true for both predialysis and dialysis patients. Attempts to modify cardiorenal risk in CKD by intensive glycemic (3) or blood pressure (4, 5) control, or combined RAAS inhibition (6–8) had modest efficacy and serious adverse events. In light of these observations, it becomes imperative to acknowledge our lack of understanding of uremic toxicity and to reexamine assumptions about biological pathways that are potentially deranged in uremia. This understanding may then satisfy a significant unmet need for better biomarkers across the spectrum of CKD. Such markers may not only be used to risk stratify patients for future clinical studies, but may also suggest targets for future pharmacological interventions.

In this report, we aim to highlight the potential role of beta-2 microglobulin (β_2_M) as a marker and possibly a mediator of some of the complications of the uremic syndrome. The classical view of β_2_M has been that the molecule is relevant to the pathophysiology of dialysis-related amyloidosis (DRA) (9–19), a truly multifactorial syndrome. The molecule itself was considered to be a relatively non-toxic uremic retention solute, whose importance as a non-creatinine (Cr) renal filtration marker was overshadowed by cystatin, when the latter was chosen for investigation in the mid-1980s and 1990s (20–22). Nevertheless, there are compelling reasons to challenge this narrow view of β_2_M.

In this paper, we will first review the basic biology and rare genetic disorders (immunodeficiency 43, OMIM #241600) associated with dysfunction of β_2_M. This overview sets the stage for reconsidering the role of β_2_M by reviewing numerous studies published in the last 5 years. In particular, we will focus on recent reports examining the role of β_2_M as a marker of renal filtration and outcomes in renal diseases across the spectrum of CKD to end-stage renal disease (ESRD) and kidney transplantation. We will also review data from non-renal diseases, a field that is usually ignored in articles focusing on nephrologists. However, this rapidly expanding literature sheds some light into the potential pathogenic role of β_2_M in human disease. Due to space limitations, we will not cover the topic of β_2_M-related amyloidosis disorders, which extend all the way from rare familial non-neuropathic amyloidosis syndromes to DRA. This is a topic that has been recently reviewed both at the biochemical (23–26) and the clinical level (10, 11), with the early literature surveyed extensively more than 10 years ago (27).

In the concluding section of this review, we will attempt to synthetize the available data, informed by our analysis of the kinetics of β_2_M and the associations between concentrations of this biomarker with outcomes. We hope that our reflections will provoke the readers to critically rethink their own assumptions about the utility of β_2_M, this easily measured, forgotten, and rediscovered protein that accumulates in renal insufficiency.

## β_2_M Physiology and Pathophysiology

**Figure 1:**
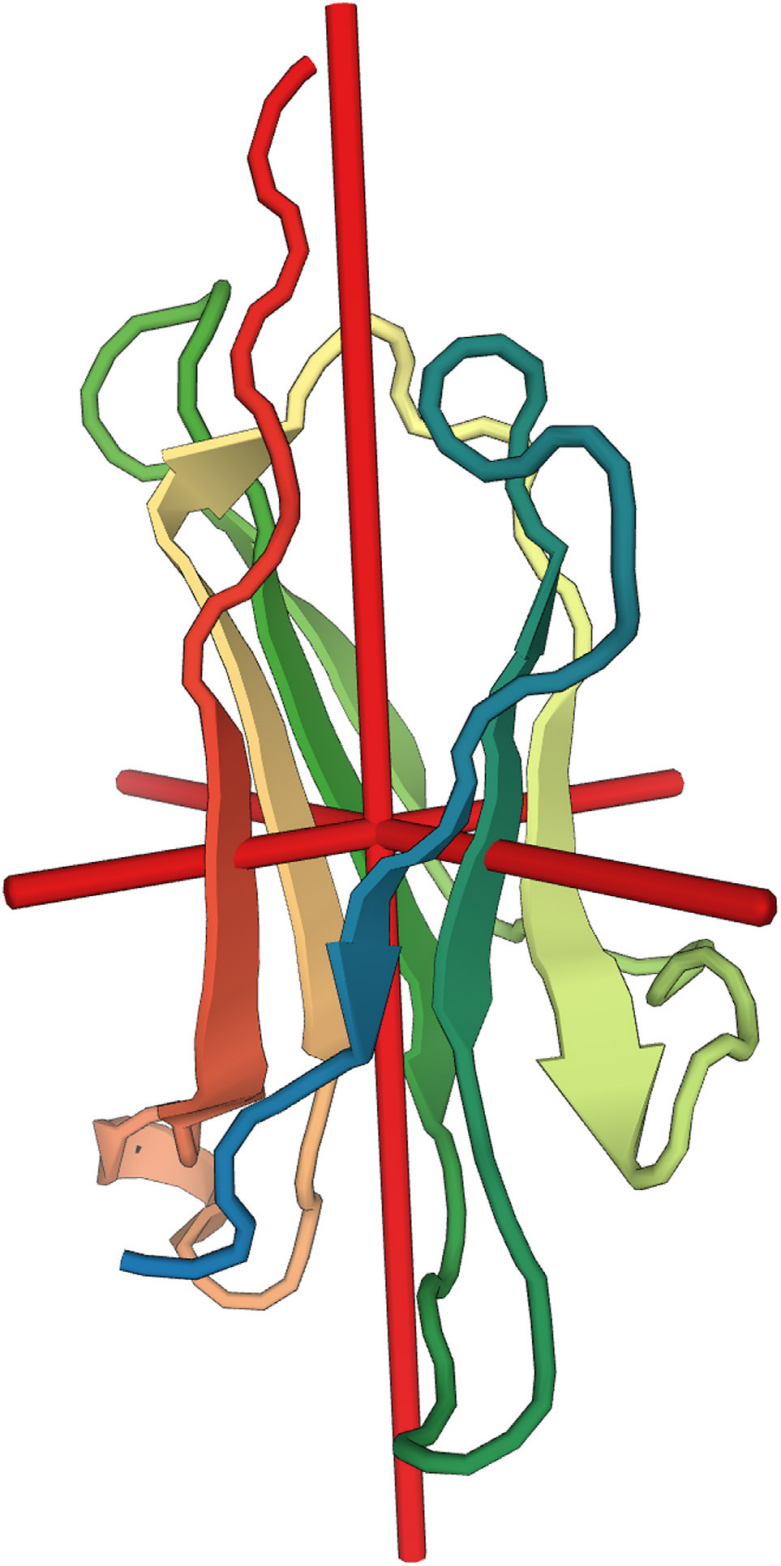
Molecular structure of beta-2 microglobulin (β_2_M). Depiction of the secondary structure of β_2_M relative to the center of gravity of the molecule (red cross). X-ray diffraction at resolution of 1.13 Å (30). Image rendered from the Protein Data Bank entry 2YXF.

Beta-2 microglobulin was first discovered in 1964 in the urine of subjects with Wilson’s disease or cadmium poisoning (28). It is a 100-amino acid protein of relatively small molecular weight (11,800 Da, size 11 Å) and it is encoded by a gene in chromosome 15 in humans. The secondary structure of the molecule consists of two large beta sheets that are linked together by a single disulfide bond (29, 30). The tertiary structure of the molecule is thus similar to the constant domain of the immunoglobulins (Figure 1). In contrast to the immunoglobulins, β_2_M does not form dimers but rather associates with the major histocompatibility complex I (MHC-I)/human leukocyte antigen I (HLA-I) on the surface of all nucleated cells. The interaction between β_2_M with the alpha chain of the HLA-I is essential for antigen presentation (31, 32). β_2_M also complexes with many non-classical MHC-I like molecules (MHC-Ib) such as CD1, MR1 (33), HLA-E, -F, -G (34, 35), neonatal Fc receptor (FcRn) (36–38), and HFE/HLA-H that are involved in mucosal immunity, tumor surveillance, maternofetal immune tolerance, immunoglobulin and albumin homeostasis as well as iron metabolism. Disorders of β_2_M function thus arise from interruption of its interaction with classical and non-classical MHC-I molecules. Their consequences can be anticipated from the normal function of the β_2_M complexes. A thorough consideration of the entire spectrum of such disorders would by necessity encompass the entire complement of classical and non-classical MHC-I molecules and it is beyond the scope of this review (see (34, 39) and (40) for a β_2_M focused survey in the field of oncology). Nevertheless, the function of FcRn merits special mention as it provides a mechanistic link between β_2_M and another biomarker of special importance to nephrology, i.e., albumin.

### The FcRn—A β_2_M-Dependent Non-Classical MHC I Molecule That Rescues Serum Proteins from Degradation

The discovery of the FcRn solved simultaneously two biological puzzles: the maternal transfer of antibodies to the offspring to protect from infections in early life and the persistence of serum albumin and immunoglobulins in the circulation (37, 41, 42). Studies in the early 1950s and 1960s demonstrated that maternal–fetal transfer of protective antibodies was dependent on the constant (Fc) part of the antibody. Furthermore, the same investigations showed that the same region underlines the long half-life of immunoglobulins vs. other proteins (~20 vs. ~5 days). The intestinal receptor responsible for transfer of antibodies from the mother’s milk to the placenta was cloned as the protein known today as FcRn (43, 44). It was subsequently shown that the same protein mediated the long half-life of immunoglobulin G (IgG) in the systemic circulation (45). Albumin also exhibits a long half-life similar to immunoglobulins, and the existence of a protection receptor had long been postulated. It was hypothesized that a similar mechanism to that of the IgG rescue underlined the protection of albumin; this hypothesis-driven research led to the identification of the FcRn as the protein that also protects circulating albumin from degradation (38).

In contrast to the classical MHC-I molecules, the FcRn lacks sequence diversity and cannot present antigens. Nevertheless, it plays an important role in mucosal and systemic sites by rescuing immunoglobulins and albumin from degradation through the transepithelial pathway (transcytosis). β_2_M interacts with the heavy chain of the FcRn and is important for its proper function (44, 46), since mice deficient in β_2_M demonstrate abnormally short half-lives of IgG (47) and sequestration of the FcRn in the endoplasmic reticulum (48). Structural and biochemical data [reviewed extensively here (37, 41, 42)] suggest that the FcRn will bind simultaneously to albumin and IgG, but at different stoichiometries: a single albumin molecule per FcRn, whereas a single IgG will simultaneously bind to two FcRn molecules. The whole body kinetics of albumin and IgG rescue by the FcRn have been studied and a simplified model developed and fit to experimental data (49). This model makes several predictions: (1) almost one third of FcRn are available for albumin recycling, (2) the maximal capacity of albumin rescue is double that of IgG, (3) the molar recycling rate of albumin is three times that of IgG, and (4) two thirds of the plasma concentration of albumin are maintained by production rather than recycling.

More recent findings [reviewed in Ref. (37, 41)] suggest a more expanded role of the FcRn as an integral part of immune defense, bidirectionally transporting immunoglobulins and antigens to the mucosal immune system. Professional antigen-presenting cells take up antigen–IgG complexes through the classical Fc receptor (FcγR) at neutral pH, thus initiating receptor-mediated endocytosis. Acidification of phagolysosomes leads to “hand-off” between the FcγR and the FcRn and the delivery of the antigen to pathways that eventually load the antigen onto MHC-I and MHC-II molecules. The end result is a potent elicitation of CD4^+^ and CD8^+^ responses against bacterial and viral antigens (37, 41).

#### The Role of the FcRn in the Kidney

The FcRn is also expressed in the kidney, where it facilitates clearance of both immunoglobulins (50, 51) and albumin (52). There is some evidence for differential handling of albumin (reclamation back into the circulation) vs. immunoglobulins (elimination into the urine) (50, 53) by the intrarenal FcRn system. The role of the FcRn in renal physiology (albumin handling) and renal disease has been explored in numerous publications involving genetic knockouts and pharmacological interventions. This was shown in experiments in which wild type were transplanted to FcRn-knockout mice and *vice versa*. Transplantation of wild-type kidney to FcRn knockout mice resulted in amelioration of albuminuria and restoration of normal urinary IgG levels. This differential handling not only prevents accumulation of protein complexes that could potentially interfere with glomerular filtration, but also provides immune protection in the urinary tract. Importantly, impaired clearance of immunoglobulins by knocking out the FcRn did not result in accumulation of IgG in the glomerular basement membrane, but rendered such animals more susceptible to nephrotoxic insults (50). There is some evidence that the podocyte FcRn system functions as an immune sensor that triggers the inflammatory response seen in certain glomerulonephritides. In particular, antithymocyte globulin treatment of podocytes increases the expression of FcRn from its high baseline state, leading to phosphorylation of the p38 mitogen-activated protein kinase, p38MAPK (54). In the same study, the percentage of glomeruli with at least two podocytes staining positive for the FcRn was characterized in human biopsies. The expression percentage was significantly higher in immune-mediated disease, including membranous nephropathy (46.7%), IgA nephropathy (66.7%), lupus nephritis (87.5%), and acute proliferative glomerulonephritis (100%), than in normal kidney samples (16.7%) (*P* < 0.05), whereas there was no significant difference between minimal-change disease and normal kidney. The relation between FcRn and p38MAPK signaling may be of pathogenetic significance since p38MAPK appears to be a major profibrotic pathway in diabetic (55), experimental nephrotic syndrome (56), and hypertensive kidney disease (57), whose inhibition leads to reduced blood pressure, sclerosis, podocyte injury, and apoptosis (58). In particular, one may postulate that activation of the β_2_M containing FcRn (e.g., by proteinuria) may trigger pathways of fibrosis inside the kidney through the p38MAPK pathway. This hypothesis, which needs to be verified experimentally, may underline the pathogenetic role of proteinuria in accelerating kidney disease progression to dialysis-dependent ESRD (59).

A major hypothesis in the nephrology literature is that proteinuria (albuminuria) underlines the progression of diverse forms of kidney disease (59, 60) and that the renoprotective effects of inhibitors of the renin–angiotensin system are partly mediated through their antiproteinuric effect (60–62). In this schema, increased oxidative stress through the NADPH oxidase system has been seen as a major contributor in promoting the progression of kidney disease (63–65), while antioxidant therapies have been proposed as a therapeutic intervention in CKD (66, 67). Interestingly, albumin overload itself has been shown to activate the renin–angiotensin system through oxidase stress and the NADPH pathway (68). These observations raise the possibility that FcRn-mediated albumin absorption may be a novel mechanism linking oxidative stress, activation of the renin–angiotensin system, and progression of kidney disease. This underexplored hypothesis has received some support in the literature. In particular, treatment with apocynin, an inhibitor of NADPH oxidase, reduced uptake of albumin by the FcRn and proteinuria in the puromycin model of nephrotic syndrome and proteinuric progressive kidney disease (69). Treatment with a monoclonal antibody against the FcRn reduced proteinuria in the same study. The possibility that the beneficial effects of renin–angiotensin inhibition are mediated to some degree through the FcRn has also been investigated in the literature. In particular, treatment of a mice anti-GBM model of glomerulonephritis with the direct renin inhibitor Aliskeren (70) reduced the glomerular deposition of IgG and reduced proteinuria in parallel with elevations in circulating IgG levels. In fact, animals that do not harbor the FcRn do not develop proteinuria and have reduced deposition of IgG compared to wild-type animals when anti-GBM nephritis is induced. The same data provided suggestive evidence that FcRn promotes the formation of subepithelial immune complex deposits (71). Finally, treatment of podocytes with IgG derived from patients with lupus, entered the cytoplasm through the FcRn to upregulate the calcium/calmodulin-dependent protein kinase IV to activate genes linked to podocyte damage and T cell activation (72). Overall, these data suggest a role for the FcRn–β_2_M complex in both normal renal handling of albumin [along with the megalin/cubilin albumin receptor (73–75)] and IgG, as an initiating event in the podocyte injury observed in many immunologically mediated renal diseases, but also the oxidative stress that appears to underlie the progression of proteinuric forms of CKD.

### Genetic Disorders of β_2_M Function

Specific mutations that interfere with the binding of β_2_M to its targets have been described in a number of conditions ranging from the rare *familial hypercatabolic* hypoproteinemia (immunodeficiency 43) (76–79) to the common *genetic hemochromatosis* (80, 81). The first two patients (siblings from a first cousin marriage) known to suffer from immunodeficiency 43 manifested a complex phenotype of hypoalbuminemia, hypogammaglobulinemia, skeletal abnormalities, and impaired delayed type hypersensitivity skin responses. These patients had circulating and total body pools of IgG less than 28% of the normal, despite having normal synthetic rates of immunoglobulins. The serum concentration for soluble HLA was less than 0.2% of normal, and iron indices were all within normal limits (79). The molecular defect was attributed to a single nucleotide trans version (G913C) in the first exon of β_2_M which impairs the function of FcRn, resulting in hypercatabolism of albumin and immunoglobulins. The immunological phenotype of β_2_M was investigated in a different consanguineous family, harboring a different homozygous splice site mutation in the first intron of the β_2_M gene (78). This mutation uncovered a cryptic splice site 4 nucleotides downstream of the canonical one, leading to a frameshift and premature termination of the β_2_M mRNA. The truncated protein had an extremely short half-life and patients had undetectable circulating and lymphocyte cell-surface β_2_M levels. HLA-I surface expression was undetectable, but there was intracellular accumulation of the HLA-A heavy chains. As anticipated, patients exhibited absence of all non-traditional MHC I molecules, i.e., CD1a, CD1b, CD1c, and FcRn from the surface of the monocytes. Similar to the first report, affected family members had severe hypoalbuminemia and hypogammaglobulinemia, with normal IgM and IgA levels. IgG responses to viral antigens were maintained, and the response to the anti-pneumococcal polysaccharide was only slightly reduced. The clinical phenotype was one of the recurrent respiratory tract infections with bronchiectasis, granulomatous dermatitis, and skin ulceration. None of the affected patients ever manifested proteinuria, possibly due to the extremely low levels of serum albumin. Circulating numbers of CD8^+^ cells were normal, but this T cell compartment consisted entirely of the γδ cells. Skin lesions were infiltrated by these T cells, autoreactive NK cells, and perforin-producing CD27^−^CD28^−^CD4 cells similar to those seen in granulomatosis with polyangiitis. The NK compartment was functionally inactivated and this prevented the development of severe autoimmune phenomena against MHC-I-deficient “missing-self” cells (78, 82).

β_2_M knockout mice recapitulate many aspects of the human disease (83) and provide a model for the effects of a severe disruption in β_2_M binding. Such mice exhibit a wide variety of immunological aberrations including suboptimal IgG responses to antigenic stimulation (84), a higher catabolic rate of IgG (47) and albumin (85), hepatic and splenic iron overload (86–89), impaired interferon gamma (IFN-γ), and other cytokine responses (90–92), higher susceptibility to parasitic (93), mycobacterial (94, 95), certain viral (90, 96) and gram (−) infections (97, 98) as well as a higher susceptibility to virus induced tumors (99, 100). This animal model has also provided controlled evidence about the rescue role of β_2_M upon serum albumin, an effect that is mediated through the FcRn (38, 85). Interestingly enough these animals do not manifest albuminuria (101, 102), a feature that is attributed to the low circulating levels of albumin in these animals or possibly the “leaky” phenotype of β_2_M knockout mice. Furthermore, β_2_M-deficient mice are in general resistant to the development of proteinuria and renal disease (101, 102). When β_2_M is knocked out in the MRL-*fas^lpr^* spontaneous lupus-like model, renal (but not skin) disease is inhibited (103, 104). Nevertheless, renal disease with the massive deposition of intrarenal immune complexes may be induced in such animals after specific and intense immunization protocols (105).

The association between β_2_M deficiency and iron overload is worthy of special mention, because it recapitulates some aspects of hereditary hemochromatosis. In the most common form of the latter disease, a C260Y mutation in the HFE molecule disrupts its association with β_2_M leading to systemic iron overload. The genetics and the clinical manifestations of hemochromatosis are very complex (106), but iron overload is seen irrespective of whether the genetic lesion refers to HFE or β_2_M. Nevertheless, there are important biochemical differences, since β_2_M-deficient mice have higher hepcidin levels which correlate inversely with the severity of hepatic iron overload (88). Furthermore, these animals fail to respond to iron overload by upregulating hepcidin levels. This may be due to abnormal cellular localization of hepcidin as seen in β_2_M silencing RNA knockdown experiments (107).

## Whole Body Metabolism and Biomarker Kinetic Model of β_2_M

Beta-2 microglobulin is continuously generated by all nucleated cells of the body. The plasma level of β_2_M is thought to reflect release of molecules that are non-covalently bound to MHC-I into the circulation and once in the plasma β_2_M is freely filtered by the glomerulus (108). β_2_M is easily and accurately measurable with most of the commercial laboratories using the highly sensitive nephelometry method (109, 110). Serum β_2_M levels are not necessarily independent of sex, race, and ethnicity (111–113). However, in all studies to date it was found that elderly have higher serum β_2_M levels. As we will see later on in this review serum β_2_M levels also increase in solid organ malignancies, lymphoproliferative disorders such as myeloma and chronic lymphoblastic leukemia, and many autoimmune diseases such as Crohn’s disease, Sjögren’s syndrome, systemic lupus erythematosus, and rheumatoid arthritis. All these are conditions, under which one would expect a higher number of cells bearing MHC molecules to be generated, or conditions in which higher shedding of β_2_M is observed (114–116).

**Figure 2:**
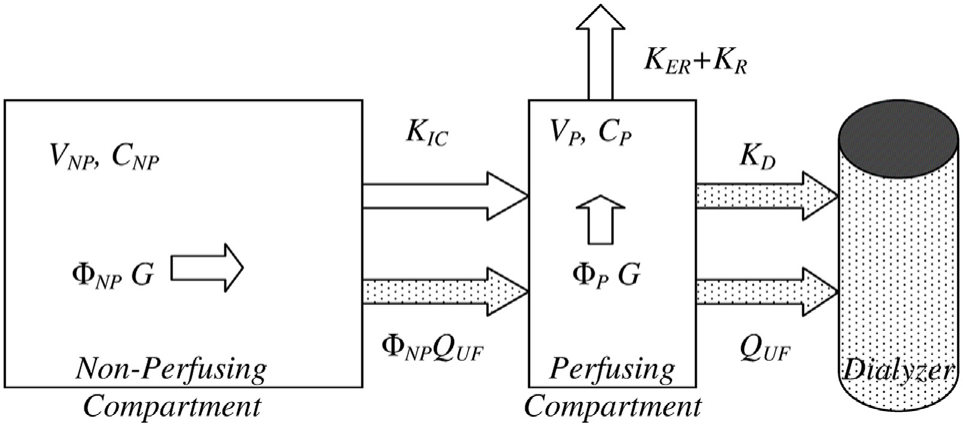
Bicompartmental beta-2 microglobulin (β_2_M) kinetics. Bicompartmental system describing β_2_M kinetics consisting of a plasma/perfusing (P) and non-perfusing/non-plasma (NP) with additional material fluxes for patients during hemodialysis sessions (stippled shapes). In each compartment, the symbols V, Φ, and C denote the absolute and fractional volume of each compartment and the concentration of β_2_M, respectively. Generation (G) takes place in both compartments, in direct proportion to their fractional volumes. *K_D_*, *K_ER_*, and *K_R_* are the dialyzer clearance, extrarenal, and residual renal clearances. Adapted from Supplementary Figure S1 of Ref. (117), reused under the Creative Commons CC BY license terms.

The multiple influences affecting both generation and elimination of β_2_M raise the need for a quantitative understanding of the factors of generation, elimination, and body compartment distribution affecting the biomarker’s concentrations. Our group produced such a *population-level* model by performing a kinetic-based meta-analysis of the existing studies in the field over the last 40 years (117). According to this model (Figure 2), β_2_M obeys bicompartmental kinetics and thus its behavior is a highly non-linear function of the relevant kinetic parameters. This is especially true in patients receiving hemodialysis (HD), who experience interdialytic (fluid ingestion) and intradialytic (ultrafiltration) compartment volume changes. The model may also be applied to study the kinetics of β_2_M in non-dialysis patients. In this case, considerable simplification is afforded by the lack of inter- and intradialytic volume changes and the discontinuous nature of dialytic clearance. A steady-state solution may in fact be recovered by solving the relevant bicompartmental system. However, this formula is too complex for practical use. This model, which is largely based on investigations in mostly Caucasian, young–middle-aged adult patients receiving HD, recapitulates many important clinical observations in both CKD and ESRD. The average serum β_2_M concentration in the “simulated” population was 1.59 ± 0.64 mg/l, while only 3.5% of simulated values were outside the upper reference range of 3 mg/l quoted in laboratory medicine references (118). This average compares favorably with the values previously reported to be: 1.53 mg/l (113), 1.62–1.86 (range of individuals with age compatible with the range in our kinetic meta-analysis) mg/l (112), and 1.9 ± 0.4 mg/l (119). Due to its derivation from first principles, this population kinetic biomarker model may also allow a more rigorous, quantitative evaluation of other factors (e.g., generation) affecting serum β_2_M concentration. This is a perspective that we explore in the conclusion of this review.

## Using β_2_M to Assess Glomerular Function

There are various ways to assess renal function with changes in glomerular filtration rate (GFR) being the most widely used method. This is achieved by assessing the plasma or the urinary clearance of filtration markers with an ideal endogenous marker being the one that appears at a constant rate in plasma, is freely filtered by the glomerulus, is neither absorbed into the circulation nor secreted by the tubules, and it is not removed from extrarenal sites. Estimation of GFR by Cr-based equations lacks precision and accuracy due to non-renal determinants—such as non-renal removal, renal secretion, and variations in muscle mass—affecting serum Cr level. Researchers have been in constant search of an ideal filtration marker. In this section, we will review the evidence arguing for the adoption of β_2_M as an additional marker of glomerular filtration in CKD.

### Role of β_2_M for the Assessment of GFR in Adults

Numerous studies to date have demonstrated large correlations between measures of renal function and suitably transformed serum levels of β_2_M (Table 1). These studies provide compelling reasons to suspect that one can estimate renal filtration with a β_2_M estimating equation. Recent research has explored the advance, if any, of such equations over the Cr-based estimated GFR (eGFR).

**Table 1:** Relationship between beta-2 microglobulin (β_2_M) and glomerular filtration rate (GFR) in adults.

Study	GFR measure	Correlation (1/β_2_M)	Correlation (β_2_M)	Slope linear regression
Vincent et al. (120)	Inulin clearance	–	–	−0.87
Wibell et al. (121)	Inulin clearance	–	−0.94	−0.89
Swanson et al. (113)	Iothalamate clearance	–	–	−0.82
Shea et al. (122)	Iothalamate clearance	0.90	–	–
Inker et al. (123)	Iothalamate clearance	–	–	−0.85
Aparicio et al. (124)^b^	^51^Cr-EDTA	0.79	–	−0.75
Grubb et al. (20)	^51^Cr-EDTA	0.59	–	–
Yun et al. (125)^c^	24-h creatinine clearance	–	–	−0.79
Jovanović et al. (126)	24-h creatinine clearance	0.80	–	–
Shea et al. (122)	24-h creatinine clearance	0.87	–	–
Aksun et al. (127)^a^	^99m^Tc-DTPA GFR	–	−0.48	–
Bianchi et al. (128)	^99m^Tc-DTPA GFR	0.76	–	−0.81
Donadio et al. (129)	^99m^Tc-DTPA GFR	0.73	–	–
Donadio et al. (130)	^99m^Tc-DTPA GFR	–	–	−0.81
Fry et al. (131)^d^	Timed urea collections	–	−0.63	–
Vilar et al. (132)^d^	Average of urea and creatinine collections	0.82	−0.72	–

*The table reports the slope of the linear regression between log concentration and log clearance*.

*– Not reported*.

*^a^Studied patients with type 2 diabetes*.

*^b^Studied patients with sickle cell disease*.

*^c^Studied patients with multiple myeloma*.

*^d^Studied patients on maintenance dialysis*.

The Chronic Kidney Disease Epidemiology Collaboration (CKD-EPI) group developed a β_2_M-based GFR estimating equation in a cohort of 2,380 patients primarily comprised of Caucasians and African Americans with a mean measured GFR (mGFR), serum Cr, and serum β_2_M levels of 47.5 (±21.7) ml/min/1.73 m^2^, 1.9 (±0.9) mg/dl, and 4.3 (±2.4) mg/l, respectively (39). β_2_M was strongly positively correlated with serum cystatin C and Cr with Pearson coefficients of 0.9 and 0.78, respectively. Serum β_2_M was negatively correlated with GFR with a Pearson coefficient of −0.85. The authors included the variables of age, sex, and race in the least error regression model for equation development—the coefficients for β_2_M were significant albeit small, similar to those of cystatin C, and smaller than those for Cr. Addition of these variables did not substantially improve equation performance in the whole cohort as well as various subgroups, therefore, the final equation did not include these variables. This report also compared the precision and accuracy of equations using the metrics of interquartile range of error (difference between mGFR and eGFR of each subject), proportion of the patients in whom the eGFR was not within 30% (1 − *P*_30_) and 20% (1 − *P*_20_) of mGFR and root mean square error, respectively (Table 2). In that cohort, the CKD-EPI β_2_M equation achieved comparable accuracy to the CKD-EPI Cr, cystatin C, and the Cr–cystatin C equation. Nevertheless, the CKD-EPI β_2_M has an advantage over the other CKD-EPI equations in that it is independent of race, age, and sex.

**Table 2:** Performance of beta-2 microglobulin (β_2_M), creatinine (Cr), and/or cystatin C-derived equations.

Equation	Interquartile range (95% CI)	1 − *P*_30_ (%) (95% CI)	1 − *P*_20_ (%) (95% CI)	Root mean square error (95% CI)
Chronic kidney disease (CKD)-EPI β_2_M	12.9 (12.2–13.8)	18.4 (16.2–20.8)	37.2 (34.6–40.1)	0.24 (0.231–0.257)
CKD-EPI Cr	11.6 (10.9–12.4)	16.4 (14.2–18.6)	34.5 (31.7–37.3)	0.224 (0.213–0.236)
CKD-EPI Cys	11.4 (10.6–12.4)	16.9 (14.9–18.6)	34.8 (32.1–37.6)	0.228 (0.217–0.239)
CKD-EPI Cr-Cys	9.3 (8.7–10.1)	11.3 (9.5–13.2)	25.5 (23.1–28.0)	0.189 (0.180–0.199)

Since the β_2_M estimating equation was not strongly correlated with age, sex, and race, the authors concluded that there are some other non-renal determinants of serum β_2_M and addition of those factors—if readily measurable—will lead to improvement in equation performance. One should note the apparent discrepancy between the lack of correction factors for age, gender, and race in the estimating equation for β_2_M and previously reported associations between these factors and the serum level of β_2_M (111, 112, 133). In multivariate adjusted models (133), only race (lower in blacks), smoking (higher in smokers), and proteinuria (higher in patients with proteinuria) retained intermediated associations with a higher serum β_2_M concentration. This discrepancy should be taken as evidence of the β_2_M to be a somewhat superior marker of renal filtration that has higher correlations to the measured GFR and smaller correlations to these non-renal determinants than Cr *per se* (123, 133). Nevertheless, the influences of non-renal determinants on other factors affecting β_2_M kinetics (e.g., generation) nullify this putative advantage, so that the overall performance of CKD-EPI β_2_M equation is not different from that of other estimating equations.

**Figure 3:**
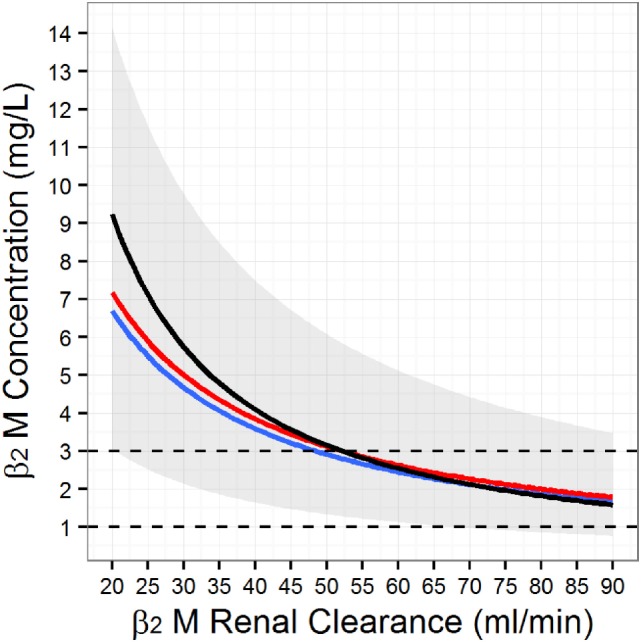
Simulated serum beta-2 microglobulin (β_2_M) as a function of the glomerular filtration rate. To generate the figure we simulated 10,000 individuals from the population model for β_2_M kinetics (117) at different levels of renal function. At each level of renal function, we computed the mean (red line), the median (blue line), and the associated population 95% range (gray band). Finally, we superimposed the Chronic Kidney Disease Epidemiology Collaboration β_2_M estimating equation (black line).

To gain a better understanding of the performance of the CKD-EPI equation (134):

$${\text{eGFR}}_{\beta_{2}\text{M}}=133\times \beta_{2}{\text{M}}^{-0.854}$$

we compared it to simulations based on our meta-analysis of the kinetic studies (117). These simulations, which were repeated for various levels of renal function, were then summarized with descriptive statistics (Figure 3), e.g., the mean (red), median (blue), and 95% quantile range (gray band). The relationship predicted by the CKD-EPI β_2_M equation (Figure 3, black) is essentially identical to the one predicted by the kinetic model, until about 40 ml/min. Below this level of GFR, the estimating equation predicts lower clearances for the same serum level of β_2_M. We can explain this divergence by considering that (a) a major underlying assumption of the kinetic model is that generation of β_2_M is not affected by renal impairment and (b) the non-renal determinants (e.g., variable generation) of serum β_2_M have been embedded into the coefficients of the estimating equation. It is worth remembering that the latter equation predicts an average relation that was estimated in cohorts with renal impairment and an average mGFR of 47.7 ml/min/1.73m^2^. If the generation rate of β_2_M varies at different levels of glomerular filtration, we would expect the statistical procedure used by the CKD-EPI investigators to balance out the influences of generation and elimination during model fitting. Furthermore, if generation is higher at lower levels of mGFR, then one would expect the CKD-EPI to provide a steeper curve between β_2_M and renal clearance than the true relation (as provided by the kinetic model) at both lower (Figure 3) and higher serum β_2_M levels. With respect to the latter point, the CKD-EPI investigators also reported that this equation underestimates mGFR at higher levels of renal function. Taken together, these observations reinforce the argument of the CKD-EPI group that poorly understood factors other than the age, sex, and race affect serum β_2_M levels. Even though the kinetic model does not allow us to pinpoint the nature of these factors, it can at least proportionate the influences of the generation and elimination processes. This feature may allow one to explore various “what-if-else” scenarios when designing clinical studies to further develop β_2_M as a biomarker.

### Role of β_2_M for the Assessment of GFR in Pediatric Populations

Creatinine clearance is known to be an unreliable marker for the measurement of GFR in children due to the changing muscle mass with age. Hence other markers, including serum β_2_M, which is not influenced by muscle mass, have been investigated as potential markers that may more accurately estimate GFR. Studies published have shown mixed results. Some studies concluded that serum β_2_M may be a reliable marker to predict GFR (135–137), while others have not (138, 139). Furthermore, the recently developed CKD-Epi-Beta Trace-β_2_M formula (123) cannot be applied in children (140). In summary, the use of serum β_2_M as a measure of glomerular filtration function does not appear to be as useful in children relative to adults. However, the urinary β_2_M excretion has been used in the diagnosis of a wide variety of renal diseases in children as we discuss in the subsequent section.

## Urinary β_2_M for the Assessment of Tubular Function

The removal of β_2_M from the serum is primarily by glomerular filtration but more than 99.9% of the filtered protein is reabsorbed and catabolized in the proximal convoluted tubule resulting in minimal urine concentration of β_2_M (usually less than 360 μg/l) (141, 142). The removal of β_2_M from the tubular fluid is postulated to be mediated through the megalin–cubilin complex (143, 144), on the basis of ligand blotting assays (145), megalin animal knockouts (146), and the human disease Donnai-Barrow/Facio-Ocular-Acoustico-Renal Syndrome (OMIM #222448) (147). This syndrome is associated with multisystemic abnormalities, developmental delay, and tubular proteinuria as a result of mutations in the *LRP2* megalin gene (148). This interaction may be mediated by the megalin component of the megalin–cubilin complex, since human cubilin mutations (megaloblastic anemia 1, OMIM #261100) manifest tubular proteinuria but with normal urinary β_2_M levels (149).

Proteins endocytosed through the megalin/cubilin complex are targeted to the endosomes, where ligands are released from their receptors through acidification (143, 150). It is not known how much of β_2_M is degraded within the lysosomes, recycled with other MHC to the membrane surface, or transported to the basolateral surface (such as thyroglobulin or retinol binding protein). Data from experiments in rats provide evidence that β_2_M is targeted to the lysosomes (151), so that degradation appears to be the most likely fate for β_2_M. However, there is also conflicting evidence for convergent apical and basolateral endocytic systems in the proximal tubule (151, 152). As human proximal epithelial cells are capable of transcytosis of the FcRn-β_2_M-IgG (153), it is possible that some of the reabsorbed urinary β_2_M is transcytosed. However, to our knowledge no study has specifically looked for transcytosis of β_2_M absorbed through the megalin pathway. Experiments reported four decades ago provide some evidence for competitive inhibition for the absorptive tubular mechanism between β_2_M and other proteins in the tubular fluid (154–157). More recent experiments suggest similar transport kinetics arguing for a single mechanism mediating this process (158). Hence, one could anticipate variable urinary excretion levels of β_2_M in the presence of glomerular proteinuria. The tubular handling of β_2_M exhibits maturation during the neonatal period. The urinary β_2_M excretion peaks by the fifth day of life and gradually declines to adult level by 3 months of age (159). This feature suggests that urinary β_2_M not only may be a reliable biomarker of tubular toxicity but also it may even have an age dependent performance.

### Role of β_2_M for the Assessment of Tubular Function in Adults

Since its initial discovery from the urine of humans with cadmium toxicity, β_2_M has been used to assess tubular function. More recently, the Nephrotoxicity Working Group of the Critical Path Institute Predictive Safety Testing Consortium assessed urinary β_2_M, along with other three biomarkers of nephrotoxicity (urinary clusterin, urinary cystatin C, and urinary total protein) in 10 mechanistic time-course studies involving 739 rats treated with eight nephrotoxins known to induce different types of renal lesions and two hepatotoxins as a means to assess specificity for kidney vs. other organ toxicity (160). Of note, β_2_M and cystatin C were specific for glomerular alternations, and with the exception of the gentamicin model, no systematic increase of either protein in urine or kidney tissue could be demonstrated when rats were exposed to other tubular toxins. Another recent animal toxicology study evaluated the performance of neutrophil gelatinase-associated lipocalin (NGAL) and four urinary biomarkers deemed acceptable by the regulatory authorities to detect acute drug-induced renal toxicity (161): β_2_M, cystatin C, kidney injury molecule-1 (KIM-1), and clusterin. In this particular study, urinary β_2_M and cystatin C increased early (prior to the detection of histological changes) and returned to the control range in the recovery phase. Furthermore, plasma β_2_M changes paralleled changes in urinary β_2_M, but correlations between the biomarker values varied according to the nephrotoxin. Nevertheless, a more extensive evaluation of 12 markers for sensitivity (renal toxicity) and specificity (non-renal organ toxicity) in 22 rat studies, reveal that β_2_M (and cystatin C) had relatively poor area under the curve (AUC) for both tubular (AUC = 0.72) and glomerular (AUC = 0.85) toxicities (162). In the same study, urinary albumin had one of the best performance for both tubular (AUC = 0.90 vs. AUC = 0.96 of the best performing KIM-1) and glomerular (AUC = 0.99, best performing) toxicity. Collectively, the recent animal toxicology data raise important questions about both the specificity of urinary β_2_M for tubular lesions, i.e., this compound appears to detect glomerular injury better than tubular damage, and its overall utility relative to the more easily obtained assay for albuminuria. Interestingly, significant correlations between urinary β_2_M and other indices of renal damage (e.g., protein/Cr ratio) have been reported in IgA nephropathy (163) and systemic lupus erythematosus (164). Both these conditions are characterized by predominantly glomerular lesions. Consequently, controlled toxicological data and observational reports suggest some caution when interpreting urinary β_2_M elevations as indicative of a bona fide tubular process. In particular, one should always entertain the hypothesis of a glomerular process leading to proteinuria and competition of the filtered protein load for the reabsorption process in the proximal tubule (156) when interpreting a high urinary β_2_M level. Needless to say, there are virtually no human data about specific diagnostic cutoffs of the urinary β_2_M; even the three aforementioned toxicological rat studies provide different cutoff values.

A meta-analysis of various urinary biomarkers has confirmed that the value of urinary β_2_M may be limited in clinical acute kidney injury (AKI) due to sepsis (165). In this meta-analysis urinary β_2_M was found to be associated with changes in serum Cr and could differentiate between prerenal azotemia and tubular necrosis, but could not predict the clinically important outcome of need for renal replacement therapy. More recently, Zeng et al. conducted a study of diagnostic accuracy in 47 patients (166). The reference test was urinary β_2_M (normal urinary β_2_M range: 230–300 μg/l). The sensitivity and specificity of urinary β_2_M in detecting tubular injury (assessed through KIM-1 staining in renal biopsies) was 86.6% and 64.7%, respectively. In summary, there is currently very limited evidence about the utility of urinary β_2_M in the diagnosis of AKI; most of the data come from the era of high-dose aminoglycoside therapy. Under these conditions, release of β_2_M in the urine may not even reflect actual toxicity (167). Important limitations of the literature to date are poor standardization of urine collection protocols for β_2_M and the poor stability of the analyte in acidic urine (168–171).

Various population-based studies have shown that urinary β_2_M levels can be used to detect tubular injury due to various toxins. β_2_M has been used as a marker of tubular dysfunction in subjects exposed to heavy metals such as cadmium with urinary β_2_M levels strongly correlating with serum cadmium levels (172–175). Rybakowski et al. showed that lithium-treated patients were more likely to have higher urinary β_2_M and lower eGFR than patients not treated with lithium (176). Beta-2 microglobinuria was also seen in HIV patients on tenofovir (177–179).

### Role of β_2_M for the Assessment of Tubular Function in Pediatric Populations

#### Tubulo-Interstitial Diseases

Urinary excretion of β_2_M either in the form of fractional excretion of β_2_M (FE-β_2_M) or 24-h urine β_2_M excretion has been used in the diagnosis of tubulo-interstitial diseases (180). This study examined children with glomerular (*N* = 114), tubular (*N* = 50), or other (*N* = 18) renal diseases and showed that children with tubulo-interstitial disease had significantly higher FE-β_2_M (mean 4.27%) compared to children with glomerular disease alone (0.104%). This difference seen was not due to impairment in GFR alone as at any given eGFR, patients with tubulo-interstitial disease had higher FE-β_2_M compared to patients with glomerular disease alone (142). Children with glomerular disease and a high FE-β_2_M who underwent a renal biopsy (*N* = 13), were found to have focal areas of fibrosis, plasma cell or lymphocyte infiltration, or tubular atrophy. These patients were found to have poorer prognosis compared to patients who had pure glomerular disease. This finding was later refuted by a subsequent study (181), which showed that the urinary excretion of low-molecular weight protein (LMWP) in children with glomerular disease did not necessarily portend a poor prognosis. Urinary β_2_M excretion has also been used in the diagnosis of a variety of renal diseases that affect tubulo-interstitial function including tubule-interstitial nephritis with uveitis (182), hemoglobinopathies such as sickle cell disease (183, 184), as well as children who have received chemotherapy as part of their cancer treatment (185).

#### Localization of Urinary Tract Infection (UTI) and Detection of Urinary Obstruction

As urinary β_2_M level is an important reflection of tubular function of the kidneys, measuring the urine level has been used in the localization of UTI in children. Studies have shown that children with upper UTI tend to have higher urinary β_2_M excretion compared to children with lower UTI (186, 187) hence allowing for more accurate localization of infection and treatment strategies. Serum and urinary β_2_M have also been shown to be elevated in children with reflux nephropathy (188).

Urinary NGAL and β_2_M have been proposed as useful tests for the diagnosis of obstructive uropathy due to ureteropelvic junction obstruction as the levels were elevated in the pre- and peri-operative period and improved with the relief of obstruction (189). However, the control group in this study was comprised of healthy children with no renal impairment, hence it is unclear whether the elevated urinary NGAL and β_2_M level was a reflection of the impaired renal function or the obstruction itself.

#### Acute Kidney Injury (AKI)

Creatinine is a poor marker for AKI due to various factors including the influence of muscle mass, fluid status, and/or delayed increase in level after the occurrence of kidney injury making early intervention impossible. β_2_M has been investigated as a candidate biomarker for AKI as it is muscle mass-independent and the rise in serum β_2_M levels occurs earlier compared to the rise in serum Cr levels (190). A recent study (191) showed that both serum cystatin C and β_2_M were better biomarkers compared to Cr in the detection of AKI in critically ill children. In a prospective study of 252 children who presented to the emergency department, urinary β_2_M, NGAL, and KIM-1 demonstrated good accuracy (AUC > 0.7–0.8) in predicting AKI (192). The caveat with using β_2_M as a biomarker for AKI is that the level varies with gestational age, hence caution will need to be exercised in using this serum marker in premature infants (193, 194).

In summary, measuring serum and urine β_2_M has been used in both predicting GFR and diagnosing renal diseases in children with variable success. Urinary β_2_M level has been helpful in the diagnosis and the monitoring of children afflicted by diseases that affect tubular function or in those who have or will be receiving medications that could affect the tubular function. Furthermore, urinary β_2_M may also be a useful marker for the early detection of AKI. Nevertheless, provocative animal and human data suggest that it may a better marker of glomerular, rather than tubular injury.

## β_2_M as a Biomarker of Adverse Clinical Outcomes and Mortality in CKD

There is limited research and evidence examining the role of serum β_2_M as a biomarker being able to predict adverse outcomes and mortality across the spectrum of predialysis CKD. We identified five recent studies examining the prognostic role of β_2_M in patients with CKD.

In the first study, the authors examined the relationship of plasma β_2_M levels to clinical and CV outcomes in 142 patients (mean age 67 years) at different stages of CKD. Plasma β_2_M levels increased with CKD stage and thus were highest in HD patients (195). Baseline plasma β_2_M levels were associated with vascular calcification but not with arterial stiffness or bone density. During a mean follow-up of 969 days, 44 patients died and 49 suffered a CV event. Higher plasma β_2_M levels were independently associated with overall and CV mortality and CV events in the whole cohort and with CV events in the predialysis cohort. Moreover, plasma β_2_M appeared to be a better predictor than well-established factors associated with outcomes in this population, such as eGFR (only for predialysis patients), inflammation biomarkers, and other factors included in a propensity score. Thus, they confirmed a strong relationship between plasma β_2_M levels and eGFR and the power of plasma β_2_M to predict overall and CV mortality and CV events in patients at different stages of CKD.

The association of serum β_2_M with hard clinical outcomes and its predictive ability was also examined in a prospective cohort study on behalf of the CKD Biomarker Consortium and the Chronic Renal Insufficiency Cohort (CRIC) Study Investigators (196). They examined the potential role of serum β_2_M as predictor of ESRD, mortality, and new-onset CV disease in 3,613 adults with CKD from the CRIC Study. During a 6-year median follow-up, 755 (21%) participants developed ESRD, 653 died, and 292 developed new-onset CV disease. After multivariable adjustment serum β_2_M was an independent predictor of ESRD, all-cause mortality, and CV events. These associations were stronger than those observed for the Cr-based eGFR (*P* ≤ 0.02).

Furthermore, in 2015 an interesting longitudinal cohort study came out by the same group on behalf of the CKD Biomarkers Consortium (197). They examined incident ESRD and mortality in 250 Pima Indians with type 2 diabetes (DM II) and whether serum β_2_M was associated with these outcomes. During a median follow-up of 14 years, 69 participants developed ESRD and 95 died. Serum β2M was associated with ESRD after adjustment for traditional risk factors and established filtration markers.

Another study examined the associations among serum β_2_M, malnutrition, inflammation, and atherosclerosis (MIA) in 312 patients with CKD between 2009 and 2015 (198). They found that serum β_2_M was more sensitive than serum Cr in predicting CV events and MIA syndrome. This study supports the hypothesis that CV events in patients with CKD should be understood as part of the MIA complex and that non-renal determinants of serum biomarkers provide prognostic information beyond that afforded by filtration biomarkers or their estimating equations.

Finally, an individual participant data meta-analysis was recently published by the CKD Biomarkers Consortium wherein they examined filtration markers, such as β_2_M, as predictors of ESRD and mortality (199). They included three general population/hazard ratio (GP/HR) studies (*n* = 17,903 participants) and three CKD studies (*n* = 5,415). They compared associations, risk prediction, and improvement in reclassification of eGFR using β-trace protein (BTP) (eGFR_BTP_) and β_2_M (eGFR_β_2_M_) alone and the average (eGFR_avg_) of eGFR_BTP_, (eGFR_β_2_M_), Cr (eGFR_Cr_), and cystatin C (eGFR_cys_), to eGFR_Cr_, eGFR_cys_, and their combination (eGFR_Cr-cys_) for ESRD (2,075 events) and death (7,275 events).

Mean (SD) follow-up times for ESRD and mortality for GP/HR and CKD studies were 13 (4), 6.2 (3.2), 14 (5), and 7.5 (3.9) years, respectively. Compared with eGFR_Cr_, eGFR_BTP_ and (eGFR_β_2_M_) improved risk associations and modestly improved prediction for ESRD and death even after adjustment for established risk factors. The authors concluded that these markers do not provide substantial additional prognostic information to eGFR_Cr_ and albuminuria, but may be appropriate in circumstances where eGFR_Cr_ is not accurate or albuminuria is not available.

In 2012, the Atherosclerosis Risk in Communities (ARIC) project investigated novel markers of kidney function as predictors of ESRD, CV disease, and mortality in the general population (200). They included 9,988 participants from population-based study in four US communities, followed for approximately 10 years. They utilized serum Cr-based eGFR calculated using the CKD-EPI equation and serum cystatin C, BTP, and β_2_M levels. The main outcomes were mortality, coronary heart disease, heart failure, and kidney failure. They found that higher serum cystatin C and β_2_M concentrations were associated more strongly with mortality (*n* = 1,425) than BTP level and all three biomarkers were associated more strongly with mortality than eGFR_Cr_ [adjusted HR for the upper 6, 7th percentile compared with the lowest quintile: 1.6 for eGFR_Cr_, 2.9 (95% CI, 2.3–3.6) for serum cystatin C level, 1.9 (95% CI, 1.5–2.4) investigators for serum BTP level, and 3.0 (95% CI, 2.4–3.8) for serum β_2_M level]. Similar patterns were observed for coronary heart disease (*n* = 1,279), heart failure (*n* = 803), and kidney failure (*n* = 130). The addition of serum cystatin C, BTP, and β_2_M levels to models including eGFR_Cr_ and all covariates, including urinary albumin-Cr ratio, significantly improved risk prediction for all outcomes (*P* < 0.001). They concluded that serum β_2_M and, to a lesser extent, serum BTP levels share cystatin C’s advantage over eGFR_Cr_ in predicting hard clinical outcomes, including heart failure. These additional markers may be helpful in improving estimation of risk associated with decreased kidney function beyond current estimates based on eGFR_Cr_. Subsequent investigations by the same group using data from the ARIC study have reported significant associations between serum β_2_M and sudden cardiac death (201) and fractures (119). Among the three biomarkers (Cr, BTP, and β_2_M)-based CKD-EPI estimating equations, β_2_M demonstrated the strongest association with sudden death [HR for fourth quartile vs. first quartile 3.48 (2.03–5.96) vs. ≤2.7 for the other kidney markers]. Renal filtration markers and albuminuria were shown to associate with fracture risk. Whereas the relationship between Cr-based CKD-EPI and risk of hospitalization for fracture was non-linear, there was a graded association between the inverse of serum β_2_M (HR per 1-SD decrease, 1.26, 95% CI, 1.15–1.37, *P* < 0.001). This risk was not attenuated and in fact increased when the investigators adjusted for the Cr-based eGFR_Cr_ to 1.37 (95% CI: 1.24–1.51, *P* < 0.001).

Therefore, when improved risk prediction (due to decreased GFR) is needed, serum β_2_M can be utilized as an alternative filtration marker beyond Cr. This finding was also independently reaffirmed in a subsequent prospective cohort study (202). In this study, the investigators sought to determine whether serum β_2_M levels have a stronger association with all-cause and CV mortality-like cystatin C compared to eGFR_Cr_ and to evaluate whether β_2_M improved risk classification beyond eGFR_Cr_, in a nationally representative sample of adults (*n* = 6,445) in the US. Both studies mentioned above were performed on samples from the *general population*.

## β_2_M in ESRD

A non-traditional risk factor for CV mortality is the accumulation and high serum levels of β_2_M (195). The interpretation of the serum β_2_M in patients with ESRD is complicated by the non-linear, bicompartmental kinetics, and large interindividual variability in kinetic parameters. This variability was recently quantified by our group in a patient-level meta-analysis of all studies reporting on kinetic parameters across the spectrum of CKD and ESRD (117). Using large-scale clinical trial simulations we showed that residual renal function is the major determinant of serum β_2_M concentrations even in patients receiving maintenance dialysis (195). Furthermore, enhanced dialytic removal of β_2_M will materially affect the biomarker’s levels only when the residual renal clearance is less than 2 ml/min. These model-derived predictions are in substantial agreement with a large body of clinical data. They also support the further development of serum β_2_M as a measure of residual renal function in patients receiving renal replacement therapy. This topic has received some attention in the recent literature with some encouraging preliminary results (132, 203). In the following sections, we undertake an extensive review of the available literature regarding serum β_2_M levels, delivered dialysis dose, method of clearance, and outcome measures. The key concepts behind the relevance of β_2_M in this field are the (a) middle molecule hypothesis [which in turn has directed the development of many of these dialysis techniques using β_2_M (204–209) as a proxy of other uremic toxins], (b) the lack of appreciation of the considerable effects of residual renal function in determining serum β_2_M levels even in patients receiving the most advanced forms of these therapies, and (c) the strong associations between β_2_M and outcomes (which rivals the magnitude of similar associations observed for other biomarkers, e.g., albumin) reported in these studies.

### Conventional Hemodialysis

Observational studies originate strong message about the predictive power of serum β_2_M (210). The prognostic implication of serum β_2_M levels for the survival of HD patients was examined in 490 prevalent HD patients divided into two groups according to their serum β_2_M levels (lower and higher β_2_M group). During the follow-up period of 40 ± 15 months, there were 91 all-cause deaths (36 from CV causes). The results demonstrated that the serum β_2_M level is a significant predictor of mortality in HD patients, *independent of HD duration, diabetes, malnutrition, and chronic inflammation*. This observational study provides a modest argument about the *clinical importance of lowering serum* β*_2_M* in patients receiving maintenance HD. Counter to this argument is a report on the relationship between serum β_2_M and survival of chronic HD patients and of the association of serum β_2_M levels with mortality (211). Surprisingly, this study showed that higher serum β_2_M levels are associated with better survival in these patients. This paradoxical association may be a manifestation of “reverse epidemiology”, since nutritional status was an independent predictor of serum β_2_M concentration in the aforementioned study.

The association of inflammatory biomarkers and β_2_M has been the focus of many studies during the 1990s before the development of modern synthetic dialyzers (17, 212–215). This partially contradictory literature suggests an association between inflammation, triggered by membrane material, and serum β_2_M concentrations. To the extent that inflammation is a non-traditional factor for CV and overall mortality, as recently reviewed by Ref. (216), one would expect the association between serum β_2_M and mortality to be partly attributed to the confounding role of inflammation. Nevertheless, there is a paucity of more modern studies examining the association between serum β_2_M and risk factors for mortality in dialysis. A small study of 40 patients in high-flux (HF) HD for more than 6 months examined the association of serum β_2_M with inflammation and dyslipidemia as CV risk factors (217). There was no correlation of serum β_2_M with C-reactive protein (CRP) and IL-6 when HF membranes were used. During the follow-up period of 3 years, 6 out of 40 patients died from CV events. A significant relationship of β_2_M with dyslipidemia and mineral bone disorders, but not with inflammation was observed. Along the same lines, other groups have reported associations of serum β_2_M with suppressed interferon-gamma production, but not the traditional inflammatory marker of CRP when patients are switched from low flux (LF) to HF dialyzers (218, 219). These observations mirror similar findings in non-dialysis-dependent CKD (198). Therefore, β_2_M might have an important role in the development of CV diseases, independent of other traditional and non-traditional risk factors even when patients are dialyzed with highly permeable HF membranes. There is a need for large, modern studies in this era of HF dialyzers and ultrapure dialyzate to better understand the magnitude and significance of β_2_M in patients receiving maintenance HD.

Observational studies and randomized clinical trials (RCTs) suggest that HF HD efficiently removes β_2_M from the blood and has positive effects on the survival and morbidity of uremic patients when compared with LF HD. The bulk of information [96% of all patients and events in the most recent meta-analysis by the Cochrane group (220)] is provided by two large multicenter RCTs. The hemodialysis (HEMO) study was a RCT designed to examine the impact of two treatment parameters (dialysis dosage based on urea Kt/V and membrane permeability) on clinical outcomes of maintenance HD patients (221). In the HEMO study, membrane flux was defined by the clearance of β_2_M (surrogate for the clearance of middle molecules). The primary analysis of the HEMO study did not show a statistically significant reduction in the rate of the primary outcome and all-cause mortality. In secondary analyses, however, a 20% decrease in cardiac death was observed for the HF group compared with the LF group. In the subgroup of patients who had been on dialysis for >3.7 years before enrollment in HEMO, HF was associated with lower all-cause mortality, cardiac deaths (221, 222), and cerebrovascular events (223). As expected, the cumulative mean predialysis serum β_2_M level during follow-up in the HF arm was statistically significantly lower than that in the LF arms. Furthermore, predialysis serum β_2_M levels predicted all-cause mortality even after adjustment for years on dialysis and residual kidney function (224). A subsequent, secondary analysis of HEMO examined the association of serum β_2_M levels and dialyzer β_2_M kinetics with the two most common causes of deaths in the HEMO study: cardiac and infectious diseases (225). In this report, the cumulative mean predialysis serum level of the middle molecule, β_2_M, correlated positively with the relative risk for infectious deaths in the HEMO study.

The Membrane Permeability Outcome (MPO) study is the second largest RCT to investigate the impact of membrane permeability on survival in incident HD patients. This study adopted a novel design, in that it specifically made a distinction between patients who had low albumin (≤ 4 g/dl) and normal albumin (> 4 g/dl) as separate randomization groups (226). The target patient population in MPO was different from the HEMO cohort, which only enrolled patients with no residual renal function who had been on dialysis for more than 3 months. In MPO, patients with serum albumin ≤ 4 g/dl had significantly better survival in the HF group compared with the LF group (227, 228). A *post hoc* secondary analysis showed that HF membranes may significantly improve survival in diabetic patients. No difference was found in patients with normal albumin levels. Our group reanalyzed the data from the HEMO and MPO studies to take into account dialyzer reuse in HEMO (reuse was not permitted in MPO). Our secondary analysis (229) reaffirmed the message from these two large, high-quality RCTs: HF dialysis with non-reused dialyzers was associated with an adjusted HR of 0.63 (95% CI: 0.51–0.78), relative to their LF counterparts. Reductions of serum β_2_M explained only one-third of the mortality benefit of the non-reused dialyzers in this report, raising the possibility that there are other, non-β_2_M mediated, beneficial effects of HF dialysis.

Other investigations have attempted to shed a light into the non-β_2_M-related effects of HF dialysis. One recent study explored the effect of membrane flux on CV risk factors and on β_2_M plasma levels in patients treated with extended dialysis (between 5 and 8 h for all patients). In this trial, patients were randomly assigned to the treatment sequences LF/HF dialysis vs. HF/LF dialysis in a crossover design after a 3-month run-in period, with each phase lasting 9 months (230). This study did not find an influence of HF filters on several traditional CV risk factors, despite the significant reduction of plasma β_2_M levels at the end of the HF phase. At the time of this writing, the beneficial effects of HF dialysis on CV outcomes can only partly be attributed or explained to reductions in plasma β_2_M levels, or even to improvements in immune function (218). Even though we do have firm evidence from the HEMO and MPO that therapies associated with more efficient dialytic removal of plasma β_2_M will improve CV outcomes, reduction in the plasma levels of this marker only partly explain this effect.

Collectively, the bulk of available evidence highlights the potential of plasma β_2_M and its higher removal to serve as biomarker of outcomes, particularly CV mortality, in patients receiving conventional thrice weekly dialysis. Pitfalls of reverse epidemiology, the less than perfect association of reduced plasma β_2_M with survival, study limitations and finally the disparate effects of plasma β_2_M in patients with hypoalbuminemia suggest that additional biomarkers are needed to both understand the effects of HF dialysis on clinical outcomes and provide a causal explanation about the role of β_2_M in mediating these outcomes.

### Hemodiafiltration

Online hemodiafiltration (OL-HDF), the most efficient renal replacement therapy, enables enhanced removal of small and large uremic toxins by combining diffusive and convective solute transport. Four meta-analyses of RCTs and narrative reviews in this area showed inconsistent results concerning the effect of convective treatments in improving general and CV survival. Nevertheless, these analyses suggest that OL-HDF may significantly reduce intradialytic symptomatic hypotension (231–236). Simulation studies anticipate that there should be a steep effect of convection volume (dose of OL-HDF) and achieved plasma β_2_M levels in patients receiving HDF (117). These simulation results originate from measurements in actual patients receiving convective therapies (237). By inference, one would expect OL-HDF to be associated with improved survival in prevalent dialysis patients receiving higher convection volume. This hypothesis is supported by observational studies and secondary analyses of RCTs. This evidence, reviewed further below, indicates that the observed reduction in mortality associated with OL-HDF correlates with the convection volumes delivered. The Dialysis Outcomes and Practice Patterns Study, an observational study involving 2,165 patients, was the first to identify the role of convection volume in patient outcome (238). This study showed that 15–25 l of substitution volume per session (not including weight loss for extracellular fluid control) resulted in a 35% reduction in mortality with high-efficiency OL-HDF relative to LF HD.

The hypothesis of an effect of convective volume on outcomes was also explored in a *post hoc* fashion in the large HDF trials reported in the last 5 years. Although the CONTRAST Study, a RCT of OL-HDF vs. LF-HD involving 714 patients was not able to prove the superiority of OL-HDF over conventional LF HD in its primary end point of mortality, *post hoc* analysis identified that larger volumes of convection fluid were associated with a significant reduction in all-cause and CV mortality (239). The Turkish HDF Study (240) was a RCT involving 782 patients which compared survival rates for OL-HDF versus HF HD; again, no significant differences in primary end points were observed, but *post hoc* analysis indicated significantly reduced mortality in the subgroup of patients receiving the largest substitution volumes (>17.4 l/session). Finally, the ESHOL Study, a prospective RCT comparing postdilution OL-HDF with HF-HD involving 906 prevalent patients, reported a 30% reduction in all-cause mortality, 33% in CV mortality, and 61% risk reduction in mortality from stroke (241). Interestingly, in this study a mean delivered convection volume of 23.7 l/session was required to achieve this magnitude of reduction in mortality.

The convection volume threshold and the range associated with survival advantage were assessed in a large cohort of incident adult patients (*n* = 2,293) treated by postdilution OL-HDF over a 101-month period (237). The relative survival rate of OL-HDF patients, adjusted for age, gender, comorbidities, vascular access, albumin, CRP, and dialysis dose, was found to increase at about 55 l/week and to plateau at 70–75 l/week. Similar analysis of predialysis plasma β_2_M concentrations found a nearly linear decrease as convection volume increased from 40 to 75 l/week. Thus, a convection dose target based on convection volume should be considered and needs to be confirmed by prospective trials as a new determinant of dialysis adequacy in patients receiving convective therapies.

An individual pooled participant analysis of the largest trials mentioned above is in line with these observations (242), suggesting a better survival when a convection volume of at least 23 l/session was delivered. Nevertheless, none of the large convective therapies trials has targeted these high volumes. Since patients were not randomized to these high targets, it is very likely that the results of these *post hoc* analyses are strongly confounded by other factors (234). In particular, high convection volumes can only be achieved if the dialysis access can support a high enough flow rate to keep the dialysis filtration fraction at a safe range (less than 30%). Participants with better functioning accesses and/or those receiving longer treatments, factors that are known to be linked to better patient outcomes, would thus have received higher convection volumes. Only well-designed RCTs with rigorous controlled convection volume targets can provide unambiguous evidence for the beneficial effects of higher convection volumes on outcomes.

### Hemofiltration

Hemofiltration is a pure convective form of renal replacement therapy, which does not utilize a dialysis component. The effect of on-line high-flux hemofiltration (OL-HF hemofiltration) vs. LF HD on mortality in CKD was studied in a small RCT (243). They compared OL-HF hemofiltration with ultrapure LF HD, assessing survival and morbidity in patients with ESRD. It was an investigator-driven, prospective, multicenter, 3-year-follow-up, centrally randomized study with no blinding and based on the intention-to-treat principle. Prevalent patients with ESRD (age, 16–80 years; vintage > 6 months) receiving renal replacement therapy at 20 Italian dialysis centers were included and centrally randomly assigned to HD (*n* = 32) or hemofiltration (*n* = 32). All-cause mortality, hospitalization rate for any cause, prevalence of dialysis hypotension, standard biochemical indexes, and nutritional status were monitored. There was significant improvement in survival with hemofiltration compared with HD (78%, hemofiltration vs. 57%, HD) at 3 years of follow-up after allowing for the effects of age (*P* = 0.05). β_2_M plasma levels remained constant in HD patients (33.90 ± 2.94 mg/dl at baseline and 36.90 ± 5.06 mg/dl at 3 years), but decreased significantly in hemofiltration patients (30.02 ± 3.54 mg/dl at baseline vs. 23.9 ± 1.77 mg/dl; *P* < 0.05). This was a small preliminary intervention study with a high dropout rate and problematic generalizability. They concluded that OL-HF hemofiltration may improve survival independent of Kt/V in patients with ESRD, with a significant decrease in plasma β_2_M levels and increased BMI. A larger study is required to confirm these results. Such a study could include an arm of higher volume OL-HDF in order to probe the differential effects (if any) of pure convection vs. mixed convection/diffusion in achieving lower plasma β_2_M levels and improving patient outcomes.

### Peritoneal Dialysis (PD)

The association of β_2_M and patient survival in patients receiving PD is underexplored. In the largest observational study to date 771 PD patients were selected from the Clinical Research Center registry for ESRD cohort in Korea in order to examine the association of serum β_2_M levels with all-cause mortality (244). The patients were categorized into three groups by tertiles of serum β_2_M levels, and the median follow-up period was 39 months. The all-cause mortality rate was significantly different according to tertiles of serum β_2_M in PD patients (*P* = 0.03). Multivariate Cox proportional analysis showed that the HR for all-cause mortality was 1.02 (95% CI 1.01–1.04, *P* = 0.006) per 1 mg/l increase in serum β_2_M after adjustment for multiple confounding factors that relate to malnutrition and for inflammation markers. However, serum β_2_M was not associated with all-cause mortality after adjustment for residual renal clearance. Even though these results are supportive of the potential role of the serum β_2_M level as a predictor of mortality in PD, they suggest that this association is a reflection of the residual renal function, a powerful predictor of mortality in patients receiving PD (245–247).

The effects of higher peritoneal clearance of serum β_2_M on mortality in PD patients are much less certain. Relevant data come from a study which investigated whether baseline peritoneal loss and clearance of albumin and other proteins is a risk factor of death (248). Mass-transfer area coefficient of Cr and peritoneal clearances of albumin, β_2_M, α_2_-macroglobulin, and IgG were calculated during a standard peritoneal permeability analysis. The total amount of albumin loss in the dialysate was also calculated. Overall mortality was studied with an intention-to-treat analysis. High baseline albumin clearance was associated with fast transport status, the presence of peripheral arterial disease, and a high comorbidity index, whereas CRP did not differ from the patients with low albumin clearance. Age, high comorbidity score, CRP > 10 mg/l, and a low serum albumin were associated with mortality. Peritoneal albumin clearances and albumin loss were not associated with death in crude and adjusted analysis. Similarly, peritoneal clearances of IgG, α_2_-macroglobulin, and β_2_M were not determinants of survival. They concluded that baseline peritoneal albumin and protein clearances are associated with signs of comorbidity, but this does not have a measurable effect on patient survival. However, these findings are tempered by the fact that higher clearances are associated with a fast-transport phenotype, which itself is a predictor of worse outcomes in PD (249, 250). Future studies should examine the effects of PD clearance irrespective of membrane transport status to better clarify the role of β_2_M and its clearance in PD.

## β_2_M in Kidney Transplantation

Chronic allograft damage is still a leading cause of graft failure 1-year posttransplantation (251). The pathophysiology of this entity is still not clearly understood but both alloantigen-dependent and alloantigen-independent factors act together to initiate inflammatory reactions that eventually lead to loss of nephrons followed by interstitial fibrosis and tubular atrophy (IF/TA) in the graft (252). Alloantigen-dependent factors that can lead to chronic allograft damage include recurrent T-cell-mediated rejection, antibody-mediated rejection, and the presence of donor-specific antibodies (253). Alloantigen-independent factors that can lead to chronic allograft damage include ischemia/reperfusion injury, donor age, arterial hypertension of the donor, drug toxicity, infections, diabetes and hypertension in the recipient, recurrent and *de novo* glomerular disease, and the presence of proteinuria.

Non-invasive diagnostic studies that may help in determining whether chronic allograft damage is present include monitoring for proteinuria, monitoring for donor specific antibodies, and monitoring for changes in the serum creatinine (254–257). But none of these tests are specific for making a diagnosis of chronic allograft damage, and elevations in serum creatinine lag behind the histological changes observed in chronic allograft damage. Thus, identifying urinary biomarkers that can detect early tubular injury would be beneficial in helping to identify those patients who need an allograft biopsy earlier on so that further progression of chronic allograft damage is prevented. Similar to the available evidence from patients with non-transplant CKD, serum β_2_M-based eGFR (<30 vs. >60 ml/min) has been found to predict CV events [HR: 2.56 (95% CI: 1.35–4.88; *P* = 0.004)], overall mortality [HR: 4.09 (95% CI: 2.21–7.54; *P* < 0.001)], and dialysis dependent kidney failure [HR: 15.53 (95% CI: 6.99–34.51; *P* < 0.001)] in allograft recipients (258). The predictive ability of elevations in serum β_2_M for subsequent allograft loss has also been reported by other groups (259). Many *de novo* donor-specific antibodies recognize free serum β_2_M (260), but the significance of this association, i.e., whether it simply reflects false-positive reactions (more likely) or it is pathophysiologically significant (less likely) remains to be established.

### Urinary β_2_M As a Biomarker in Chronic Allograft Damage

Proteomic analysis has been used in various studies in an attempt to identify a protein biomarker pattern that can help reveal chronic allograft damage. A very promising approach (261) used surfaced-enhanced laser-desorption/ionization time-of-flight mass spectrometry (SELDI-TOF-MS) to identify urinary proteins as biomarkers for chronic allograft damage. In this retrospective study, there were 34 renal transplant patients (disease group) with histologically proven chronic allograft damage, with an eGFR less than 45 ml/min who were more than 1-year posttransplantation. These patients were compared to a “control” group of 36 renal transplant patients with normal renal function (eGFR > 50 ml/min). Significantly higher concentrations of β_2_M were observed in the urine of the patients with chronic allograft damage compared with the controls (261). In another study (262), using the same population as that in Ref. (261), OrbiTrap mass spectrometry was utilized to analyze the urine further for identification of more biomarkers specific to chronic allograft damage. Again β_2_M was shown to be significantly increased in chronic allograft damage, with an approximately 50-fold increase of β_2_M expression in this cohort compared to the control group (*P* < 0.0001). Other proteins that were significantly increased in the chronic allograft damage cohort were clusterin and NGAL. Apolipoprotein A1 and uromodulin levels were significantly decreased in the same cohort compared to the control group (262).

Despite these encouraging observations, we currently lack a firm understanding of the pathophysiological processes underlying chronic urinary β_2_M elevations in kidney transplant recipients. One possibility is that they reflect chronic immunological injury. This is certainly possible, since urinary β_2_M is increased in patients with *acute rejection*. A previous study using unbiased proteomic analysis (SELDI-TOF-MS) identified many urinary fragments in the mass/charge (m/z) region 5,270–5,550 (region I; five peaks), 7,050–7,360 (region II; three peaks), and 10,530–11,100 (region III; five peaks) that always occurred together; the normal urine pattern had no peak clusters in these m/z regions (263). Interestingly, about 18% of patients with stable allograft function exhibited this pattern. A follow-up investigation by the same group used liquid chromatography–mass spectroscopy techniques to identify these peaks as cleaved β_2_M. The authors concluded that fragmented urinary β_2_M can serve as a potential biomarker for acute tubular injury due to rejection in renal allografts (264). The association of urinary β_2_M with acute rejection has been noted in two other unbiased proteomic studies utilizing matrix-associated laser desorption ionization time-of-flight mass spectroscopy (265, 266) by the same group. Whereas the first study suggested specificity for acute rejection, the second one did not, as β_2_M elevations were also seen in patients with non-transplant forms of CKD. Interestingly, another group reported that urinary β_2_M is elevated in renal transplant recipients even in the setting of good allograft function; this was different from patients with non-transplant-associated CKD who had high urinary β2M levels only when the Cr clearance was less than 30 ml/min/1.73 m^2^ (267). One could postulate that elevations in urinary β_2_M in the absence of changes in serum Cr could be used to detect acute rejection early. Furthermore, chronic elevations in urinary β_2_M could reflect ongoing low-grade immunological injury, leading to IF/TA and eventually to allograft loss. Larger studies are needed to obtain a better understanding of the factors affecting urinary β_2_M and its determinants in renal transplant recipients.

In summary, urinary β_2_M may be sensitive for this entity, but further research in this area is needed to identify whether it can be used as a reliable biomarker for identifying patients with early chronic allograft damage due to immunological factors who need an allograft biopsy for the effective management of this complex disease process.

## β_2_M in Non-Renal Diseases

Not only the pathology related to renal disease but also non-renal etiologies have influence on serum β_2_M level. Higher serum β_2_M level can be seen in patients smoking, of non-black race, and with a higher amount of protein excretion in the urine (133). As β_2_M is a light chain subunit of MHC class I antigens, it is present in all nucleated cells, especially on immunocompetent cells such as macrophages, active T and B lymphocytes. During normal cell turn over, it is released into the body fluids. Pathologies with high cell turnover, such as hemato-oncological conditions, and rheumatologic diseases are associated with higher serum β_2_M levels (268, 269). As many of these conditions may be associated with the subsequent development of CKD (270–272), we will briefly review some of these associations. Our aim is not to compile an exhaustive presentation of this large and rapidly expanding literature. Rather, we aim to draw attention to representative reports from other areas of Internal Medicine and highlight the relevant key messages for Nephrologists.

### Hemato-Oncological Pathology

In hematological malignancies, such as leukemia, lymphoma, and multiple myeloma, serum β_2_M level is found to be elevated, despite preserved renal function. It has been reported that 60% of patients with mantle cell lymphoma have high pretreatment serum β_2_M level (273). This elevated value is independently associated with unfavorable prognosis of most of the hematological malignancies (269, 273–277). These associations persist despite adjustment for well-validated clinical prognostic scores and therapy indicators (274, 278, 279).

In multiple myeloma, serum β_2_M level is the main determinant of the International Staging System (ISS, Stage I: β_2_M < 3.5 mg/dl and albumin > 3.5 g/dl, Stage II: β_2_M > 3.5 but less than 5.5 mg/dl or β_2_M < 3.5 mg/l and albumin < 3.5 g/dl, Stage III β_2_M > 5.5 mg/l). β_2_M predicts not only the prognosis but also the progression of asymptomatic disease (HR 3.30; *P* = 0.002) (280) and even outcomes after stem cell transplantation (281, 282). The association between serum β_2_M and albumin upon patient prognosis in myeloma, not only is reminiscent of similar associations noted in the dialysis literature but also is very robust statistically. Even recent proposals for a revised ISS (283) based on emerging biomarkers (e.g., chromosomal abnormalities) or the levels of soluble free light chains (284) have highlighted the prognostic significance of high serum β_2_M levels. Although the association between β_2_M and prognosis has been interpreted to reflect a higher tumor burden in myeloma (285) or a more aggressive (286, 287) myeloma subtype, it is important to realize that renal insufficiency may underline at least in part the higher levels of serum β_2_M seen in this disease (288). Despite the different cell origin, chronic leukocytic leukemia recapitulates the findings from multiple myeloma. β_2_M is a well-recognized adverse prognostic factor in this disease (289–291); even though one would expect the utility of this marker to be higher at the latter than the earlier stage of this disease, this hypothesis is not entirely borne out by observations (290). Even patients with early-stage disease have elevated serum level of β_2_M, which may reflect more aggressive behavior of the malignant process. This alternative hypothesis, i.e., that serum elevations of this biomarker may reflect the combination of higher tumor burden and more aggressive biology, is supported by observations that higher serum levels of β_2_M are associated with shorter time to therapy (291). Furthermore, failure to normalize serum β_2_M after 6 months of kinase inhibitor therapy (ibrutinib) was associated with inferior progression-free survival [HR 16.9 (95% CI: 1.3–220.0), *P* = 0.031] for ibrutinib-treated patients. This association persisted after multivariate adjustments (292).

Elevated serum β_2_M level can also be seen in patients with solid cancers, such as ovarian cancer (293), gall bladder cancer (294), prostate cancer (295), breast cancer (296), and renal cell carcinoma (297). Its higher value is closely related to the poor prognosis and aggressive characteristics of the tumor (293–297). Due to the high prevalence of high β_2_M in patients with ovarian cancer, β_2_M has been incorporated into the FDA approved OVA1 multianalytes assay for risk stratification of adnexal masses (298, 299). OVA1 measures the serum levels of five analytes, CA125, transthyretin, apolipoprotein A1, transferrin, and β_2_M. Results are reported as high or low risk for ovarian cancer and are used to determine whether referral to gynecologic oncology is required prior to surgical treatment of an adnexal mass.

More recently, evidence has emerged that implicates serum β_2_M level as a global biomarker of occult malignancy [HR: 1.25 (95% CI: 1.06–1.47; *P* = 0.002 for the trend of higher risk with increasing β_2_M quartile)], and more narrowly colorectal cancer risk [HR: 2.21 (95% CI: 1.32–3.70; *P* = 0.001 for the trend of higher risk with increasing β_2_M quartile)]. These associations, which were not attenuated after adjustment for an inflammatory biomarker, CRP, or even renal function (eGFR) of 12,300 patients, were noted in the prospective ARIC study. Significant associations were also observed for mortality from total, lung, and hematological cancers (300).

### Autoimmune Disease

Serum β_2_M is elevated in autoimmune diseases as well. Higher serum levels are seen in patients with systemic lupus erythematosus and adult-onset Still disease, especially in those with active diseases and hemophagocytic syndrome (268, 301). After therapy, the serum β_2_M level decreased significantly (268). Urinary β_2_M has been shown to correlate with overall and renal disease activity scores and proteinuria (164). Patients with active primary Sjögren’s syndrome, notably those with increased systemic disease activity (302) and history of lymphoma (303, 304), and hemophagocytic lymphohistiocytosis (305) were also found to have elevated urinary β_2_M level. Interestingly, higher levels of *urinary* β_2_M are found in patients with active primary Sjögren’s syndrome and impaired eGFR (306).

## β_2_M: Synthesis and the Way Forward

In this review, we examined the recent literature linking elevated serum circulating and urinary β_2_M levels to outcomes across the spectrum of renal impairment and also its role as biomarker in non-renal diseases. This literature suggests that β_2_M may be a particularly strong (sensitive) biomarker for both morbidity and mortality across numerous clinical conditions. This lack of specificity for particular clinical states, necessitates the application of a suitable context that would allow the interpretation of alterations in serum and/or urinary β_2_M levels. Such a framework would by necessity be context specific given the ubiquity in expression of β_2_M. For future applications in non-dialysis-dependent CKD, such a model would most likely have to incorporate additional biomarkers to derive a complex, multivariate measurement of renal function. Existing approaches such as the combined β_2_M and BTP formula referenced previously (199) show one possible research thread that may yield fruitful results. Nevertheless, it is worth remembering that the concentrations of several LMWP’s retained in CKD are poorly predicted by different eGFR formulas in a CKD population (stages 2–5 not on dialysis) (307). If this is indeed the case, then one may not improve much upon existing estimating equations through simple formulas based on biomarker level averaging, e.g., as was done when developing the cystatin-C/Cr eGFR (308) and β_2_M-BTP formulas (123). This raises the question of alternative approaches for the full subsequent development of β_2_M as a biomarker in CKD.

We believe that the way forward for β_2_M should be based on quantitative models for generation (non-renal determinants) and elimination (renal determinants) of this biomarker. For example, the population kinetic model we put forward (117) for the exploration of the “middle molecule” hypothesis for uremic toxicity maintains the separation between the processes of generation and elimination, while generating predictions that verify clinical observations in the dialysis population. When applied outside its intended application domain, i.e. in the field of CKD, it generates predictions that should be contrasted against the relationship between plasma β_2_M and the measured GFR estimated from extensive database analyses. Hence this model, developed on fewer than 150 patients, who were nonetheless extensively phenotyped, draws attention for further research on the generation (non-renal determinant) mechanisms that affect the serum levels of β_2_M.

Future research should expand this model to account for changes in the concentration of β_2_M in the urine. Such studies are urgently needed, because the urinary β_2_M appears to have a much larger utility than previously recognized. Animal toxicology experiments conducted under rigorously controlled conditions (160, 162) and provocative observations in glomerular diseases (163, 164) suggest that urinary β_2_M may in fact be a better marker of glomerular than tubular damage. This finding, backed by controlled experiments in hundreds of animals and observations in a much smaller number of humans, seems to go against textbook dogma. The latter, however, is based on a handful of observations (20 normal, 15 with glomerular, and 15 with tubular pathology due to cadmium poisoning and hereditary syndromes) made 50 years ago at the dawn of the clinical chemistry and renal pathology and before the complex β_2_M-dependent mechanisms of protein transport in the kidney were deciphered (141). It is high time that studies acknowledging the biology of β_2_M and its complex compartmental kinetics in serum and urine are undertaken so that the role of β_2_M as a biomarker be clarified. The outcomes of this exercise are not merely academic and are not limited to the field of Nephrology, considering the importance of β_2_M in other fields (mainly hematological oncology). Furthermore, this re-examination has the potential to link β_2_M to another important biomarker, albumin whose kinetics and biofluid levels are controlled by β_2_M through the FcRn.

At this point, we would like to put forward a hypothesis that we think ties together many observations in both CKD and ESRD: the emergence of non-renal processes as determinants of serum β_2_M levels as renal function declines and the beneficial effects of HF-HD dialysis in patients with hypoalbuminemia as highlighted by reports from our group (229, 309). We hypothesize that this higher generation comes not from high cell turnover, e.g., as in oncological conditions, but from altered cell binding of β_2_M to the many proteins that it chaperones. According to the model, the level of renal function is the main determinant of plasma β_2_M concentrations by affecting both removal (glomerular filtration) and generation of free β_2_M. Interference with the binding of β_2_M to MHC and non-classical MHC molecules [possibly in the endosomes where these interactions are initiated (310, 311)] by other uremic retention solutes constitutes a major source for the heightened generation of β_2_M in uremia. Clinical manifestations of these alterations result both from the higher concentrations of β_2_M (e.g., DRA) as well as the altered MHC/non-classical MHC function, i.e., the phenotype of β_2_M deficiency highlighted in the β_2_M knockout mice. Observations in these animals parallel the clinical observations and laboratory associations in patients with renal dysfunction: dysregulated IFN-γ production (312, 313), tuberculosis (314–316), acute infections, and suboptimal antibody responses [all reviewed in Ref. (317)]. Patients with renal dysfunction or on dialysis have also a higher incidence of tumors that are considered to be of viral origin in registry studies (318–323). According to this model, the hypoalbuminemia seen in many dialysis patients is a reflection of a widespread abnormality in albumin rescue through the FcRn.

This hypothesis could provide an explanation for the beneficial effects of HF-HD in hypoalbuminemic patients. Although suggested by some *in vitro* studies (324, 325), other *ex vivo* (326, 327) and *in vivo* (328) investigations did not demonstrate an effect of flux on β_2_M gene transcription or protein expression. On the other hand, both *in vitro* (329) and *in vivo* (328) flow cytometric studies have shown that dialysis with LF membranes is associated with a larger dissociation of β_2_M from the HLA-I complex compared to their HF-HD counterparts. To the extent that this model is true, it would provide a partial molecular explanation for the clinical associations between higher β_2_M concentrations (greater disruption of β_2_M binding) and infectious mortality (210, 224, 225). This model yields hypotheses about the binding behavior of β_2_M to its targets, and the resultant regulation of biological processes, e.g., IFN-γ and immunoglobulin levels in relation to higher clearance (renal or dialytic), which can tested in small randomized crossover studies and laboratory experiments (218, 313, 329, 330). In that regard, a “candidate toxin” approach based on the EUToX Uremic Solutes Database (331) could provide a way to test a number of known toxins *in vitro* experiments for a disruptive effect on the protein complexes of β_2_M. Given the potent effect of inflammatory stimuli on the function of the MHC/β_2_M system, future work in this area should attempt to control for the confounding effects of microinflammation, which is prevalent in dialysis patients, as well as possible dialysis membrane–immune system interactions.

## Conclusion

In conclusion, β_2_M is a promising marker to assess glomerular and tubular function in adults. It has similar performance to the Cr-based estimating equations as a measure of renal function, but may be more strongly associated with CV morbidity and mortality than Cr, or other small molecular renal filtration markers. β_2_M is also an important, emerging biomarker in numerous non-renal diseases. Plasma and urinary β_2_M levels can be reliably and cost effectively measured, which makes it an ideal screening tool. Plasma and urinary β_2_M levels can increase in certain conditions, which might limit its efficacy as a diagnostic marker in these populations. Future studies should be undertaken with the aim to link alterations in plasma and urinary β_2_M levels to its renal and non-renal determinants and also to the levels of albumin, which is regulated by the complex of the β_2_M–FcRn.

## Author Contributions

Each author was assigned a particular subtopic within this review. They researched the literature independently and provided their contribution to the corresponding author who integrated the manuscript. CA: conceived the paper idea, researched the biology and genetic disorders of β_2_M, animal toxicology studies, the kinetic model, and compiled the manuscript. SSC: researched the literature about non-renal diseases. Y-HN: researched the pediatric nephrology literature. M-ER: researched the literature of renal replacement therapies and the outcome studies in CKD. She also generated the first draft of the manuscript. KS: researched the literature on the use of the biomarker for glomerular and tubular disorders. PS: researched the literature on renal transplantation. AT critically reviewed the manuscript and drafted the response to author comments. All the authors were responsible for reviewing the final version of the article that was submitted to the journal.

## Conflict of Interest Statement

The authors declare that the research was conducted in the absence of any commercial or financial relationships that could be construed as a potential conflict of interest.
